# Beyond survival: microbial dispersion via aerosolization as an evolutionary trait

**DOI:** 10.3389/fmicb.2026.1923366

**Published:** 2026-08-20

**Authors:** Emily A. Kraus, Adam Gillison, Rocio Rodriguez, Margot M.-F. Thomas-Gatel, Sam Golon, Carolyn R. Cornell, Jane E. Stewart, Amaya Garcia Costas, Stephen D. J. Archer

**Affiliations:** 1 Department of Environmental Engineering, University of Colorado Boulder, Boulder, CO, United States; 2 Department of Chemical and Biological Engineering, Colorado State University, Fort Collins, CO, United States; 3 Department of Agricultural Biology, Colorado State University, Fort Collins, CO, United States; 4 Department of Biology, Colorado State University Pueblo, Pueblo, CO, United States; 5 Department of Microbiology, Immunology, and Pathology, Colorado State University, Fort Collins, CO, United States; 6 Bioeconomy Science Institute – AgResearch Group, Grasslands Research Centre, Palmerston North, Manawatū, New Zealand

**Keywords:** atmospheric microbiology, bioaerosol, biogeography, differential aerosolization, ecosystem connectivity, microbial physiology

## Abstract

Airborne dispersion of microorganisms is a constant ecologically significant global process. However, the initial stage of this process, the uplift of microbes to the atmosphere, remains poorly understood as an ecological filter. Differential aerosolization could serve as a potent selector allowing a subset of microorganisms to disperse via air more efficiently, providing potential advantages in establishment in new environments. While traits associated with atmospheric survival and deposition are well documented, microbial aerosolization is still generally presumed to be stochastic, primarily due to the small size of microorganisms and their lack of active biological ejection mechanisms like those found in seeds and larger fungal spores. However, emerging evidence suggests that uplift into the atmosphere is a dynamic interaction between physical forces in the environment and specific biological traits. This review synthesizes observations from genomic source tracking studies and laboratory experiments that describe how preferential enrichment of certain taxa into the atmosphere is based on intrinsic properties including extracellular polymeric substance (EPS) mediated aggregation, cell surface hydrophobicity, surfactant production, and other potentially relevant microbial traits. Additional candidate traits that may contribute to enhanced aerosolization are identified along with the potential mechanistic basis by which they might influence uplift. Future work with controlled chamber studies on single organisms and integration of atmospheric flux measurements with trait-based microbial uplift can provide a mechanistic basis for more accurate models of bioaerosol flux. Improving our comprehension of bioaerosol aerosolization behavior and flux is critical to understanding the dispersal of microorganisms across diverse habitats and their subsequent impacts on ecosystems, global climate, and the spread of diseases.

## Introduction

### Microbial aerosolization mechanisms

**Figure 1:**
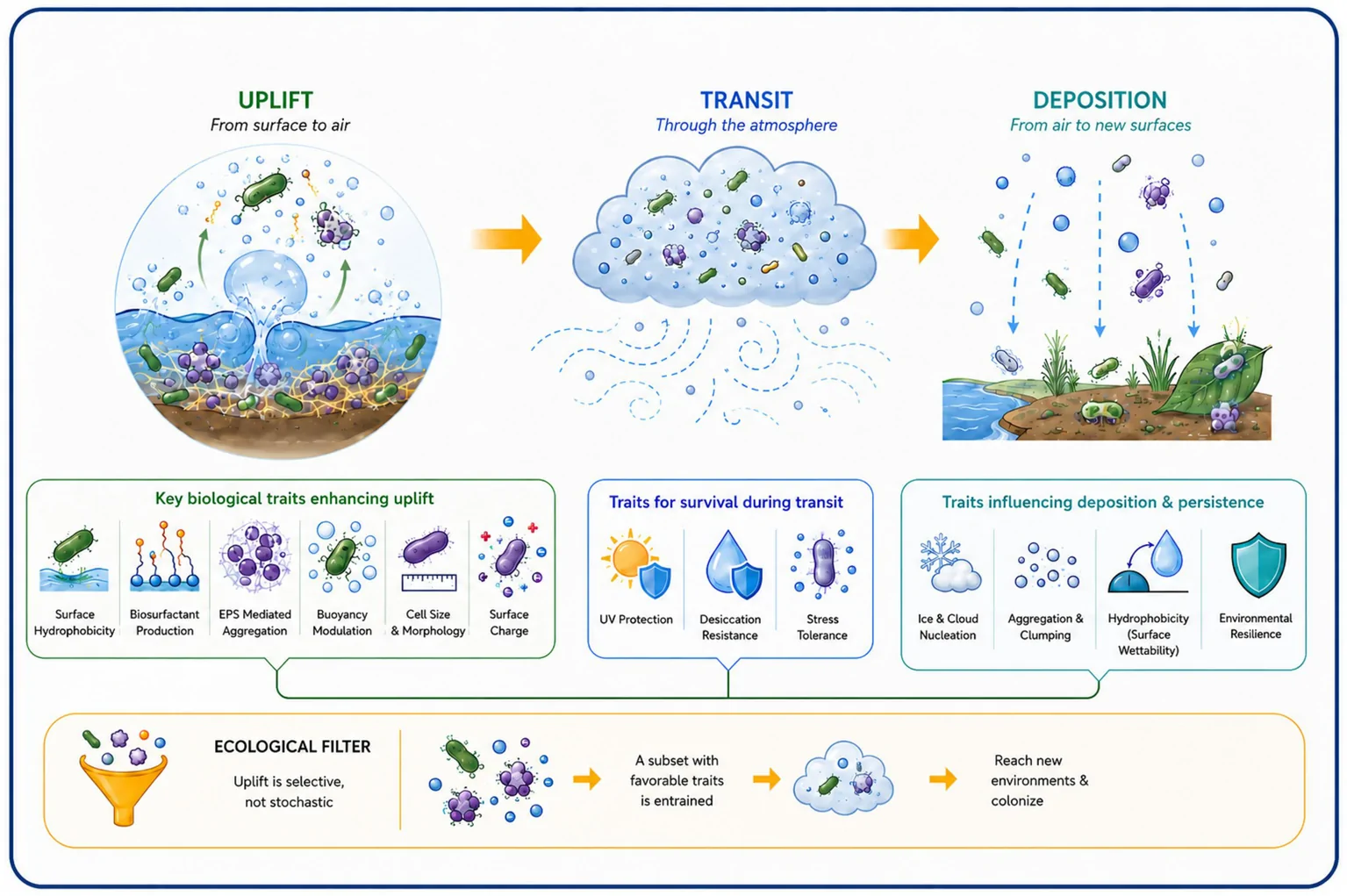
The microbial journey to the atmosphere. Although the main focus of airborne microbial transport has focused on the microbial mechanisms related to transit survival, we propose the greatest opportunity for selective pressures lies in the differential ability for the initial phase of transportation, uplift. Understanding and quantifying trait-mediated microbial aerosolization will improve predictions of bioaerosol fluxes and their impacts on ecosystems, climate and human health.

The aerobiome constitutes a globally connected, continuously exchanged microbial reservoir that plays a central role in ecosystem connectivity, biogeochemical cycling, and disease transmission. This microbial habitat is sustained by an estimated emission of ~10^24^ cells aerosolized from land and aquatic environments annually (Burrows et al., 2009). Although microbial dispersal via the atmosphere is conceptually divided into three phases of uplift, transport and deposition, most microbial ecology studies typically focus on the transport phase (Figure 1) (Smets et al., 2016; Tignat-Perrier et al., 2019; Després et al., 2012). This airborne transport phase is generally considered purely a medium through which microorganisms may survive multiple environmental stressors through traits that protect them, primarily from UV exposure and desiccation (Després et al., 2012; Amato et al., 2017; Morris et al., 2025). In contrast, the initial aerosolization step and associated microbial traits remain comparatively unexplored despite representing a key ecological filter wherein only a small non-random subset of microorganisms is successfully entrained into the atmosphere. Unlike fungi which actively eject spores, microorganisms lack specialized dispersal structures and have traditionally been assumed to rely on stochastic physical perturbations such as wind shear for uplift. However, increasing evidence from laboratory simulations and the coupled sampling of air and source microbiomes indicate that preferential aerosolization is substantially driven by microbial properties rather than the physical mechanism of aerosol generation (Michaud et al., 2018; Archer et al., 2023).

This perspective reframes aerosolization as a potential target of selection where traits that enhance the probability of uplift may be indirectly favored when atmospheric dispersal enhances fitness. Studies have shown that specific bacterial and fungal taxa are enriched in air compared to underlying soil (Archer et al., 2023; Dressaire et al., 2016), plant (Lymperopoulou et al., 2016), and seawater communities (Michaud et al., 2018; Fahlgren et al., 2015; Rastelli et al., 2017). Observations of preferential aerosolization of pathogenic bacteria from pool water (Angenent et al., 2005) and anaerobic digesters (Moletta-Denat et al., 2010) suggest enhanced partitioning into an aerosol phase may be an adaptive colonization strategy for disease transmission. Laboratory aerosolization experiments with multiple bacterial strains and nebulization methods found enhanced aerosolization was dependent on bacterial properties rather than the aerosol generation mechanism or cell size/shape (Perrott et al., 2017). Furthermore, traits inferred from 16S ASV taxonomic assignments such as larger cell size, colony formation, fermentation capability, and spore formation have recently been associated with enhanced uplift potential (Echenique-Subiabre et al., 2025).

**Figure 2:**
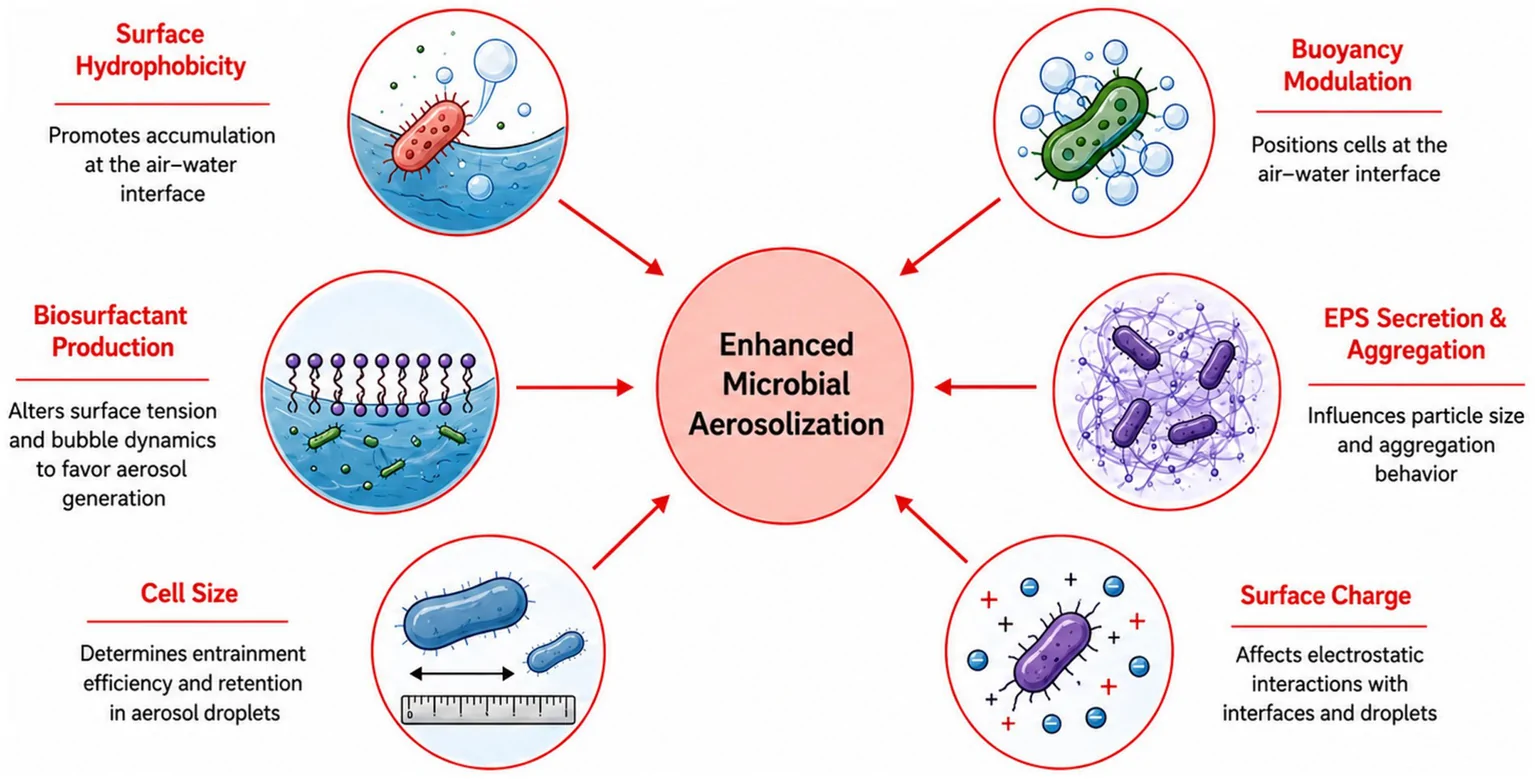
Potential biological traits that could enhance microbial aerosolization over aquatic and terrestrial landscapes. Broadly, these can be separated into traits that increase surface interaction or reduce the energy required for organisms to be released from surfaces into the air creating a higher probability for lofting.

Analogous to dispersal strategies in macroorganisms (e.g., specialized seed structures of plants), microbial traits may enhance access to the atmosphere and subsequent airborne dispersal (Figure 2). Surface physiochemistry, extracellular polymeric substance (EPS) production, and particle aggregation can promote aerosolization by modulating interactions with physical uplift processes. For example, EPS can facilitate buoyant aggregate formation with entrained bubbles in aquatic systems, increasing residence time at the air-water interface and the probability of aerosolization (Dervaux et al., 2015). Deposited, desiccated EPS matrices can form brittle films that fragment into readily aerosolized residual biomass, potentially facilitating short to mid-range dispersal. Such mechanisms have been implicated in microbial dispersal in extreme environments such as the Dry Valleys of Antarctica, where extracellular material appears to contribute to microbial spread (Wood et al., 2008). Collectively, these observations challenge the longstanding question in microbial ecology of whether “everything is everywhere” (Baas Becking, 1934), instead suggesting that access to the atmosphere is a selective barrier determined by both microbial traits and physical aerosol generation mechanisms. At cellular scales where intrinsic aerodynamic differences between cells are minimal, traits such as adhesion propensity, aggregation, and other extracellular properties likely exert some control over aerosolization probability. Framing uplift as an evolutionarily relevant process thus shifts it from a purely physical phenomenon to a biologically mediated filter, underscoring the need for integrative approaches that combine microbiology and atmospheric science with controlled experimental systems and field-based flux measurements to resolve mechanisms governing microbial dispersal. Considering preferential aerosolization as an evolutionary advantageous trait can improve our ability to predict microbial movement, disease exposures, and ecosystem impacts in a rapidly changing world.

## Microbial traits influence aerosolization

### Cell surface hydrophobicity enhances uplift from the sea surface

Sea spray aerosol (SSA) is a dominant source of ocean-derived microorganisms in the atmosphere wherein their transfer from seawater to air is governed by selective biological and physical processes (Cunliffe et al., 2013). SSA is produced by bubble bursts at the air-water interface in the upper 1 mm of the ocean, termed the sea surface microlayer (SML), which is highly enriched in organic matter and microorganisms compared to underlying sea water (Blanchard and Syzdek, 1982; Aller et al., 2005; Rastelli et al., 2017). This enrichment in the SML propagates into the selective transfer of certain microorganisms to SSA, resulting in over-representation of bacterial community members that are less abundant in seawater (Rastelli et al., 2017; Aller et al., 2005). This selective aerosolization is linked to differences in microbial cell surface hydrophobicity, often imparted by a waxy coating or lipid-envelope, which facilitates the cell’s movement to the SML via bubble scavenging (Cunliffe et al., 2013). For example, gram-positive Actinobacteria, particularly those with a waxy, mycolic acid coat like *Corynebacteriales*, are often enriched in SSA while closely related strains that lack these envelopes (e.g., *Corynebacterium kroppenstedtii*) are consistently under-represented in SSA (Michaud et al., 2018). Similarly, viruses also display differential transfer into SSA based on presence of a hydrophobic lipid envelope (Michaud et al., 2018). Lipids have been identified as the most strongly enriched organic component in artificial SSA, reaching concentrations up to 140,000 times greater than seawater and further supporting the importance of lipid-mediated hydrophobic surface interactions in bubble-driven aerosol generation (Rastelli et al., 2017). Though microbial dispersal from oceans via SSA is an area of active research, these observations indicate that the associated microbial flux is dictated, at least in part, by cellular surface properties, primarily hydrophobicity, leading to the selective transfer of microorganisms and a microbial community in the air that is distinct from the underlying seawater. By increasing residence time at the air-water interface of the SML, such traits may enhance the probability of atmospheric entrainment and provide a potential dispersal advantage in vast marine environments. Although these features likely evolved under selection for other functions (e.g., membrane structure), they may have a secondary role facilitating airborne dispersal and colonization.

### Biosurfactants alter bubble burst dynamics

Biosurfactants are amphipathic metabolites composed of coupled hydrophobic and hydrophilic moieties which allow these molecules to align at air-liquid interfaces. Biosurfactant secretion is a plausible microbial mechanism for enhancing aerosolization since the phase boundary alignment of biosurfactants reduces interphase surface tension, consequently altering bubble formation and rupture dynamics. This is particularly significant since, as described above, bubble bursts play a significant role in bioaerosol generation in aquatic and wet surfaces in terrestrial environments. The production of many biosurfactants is regulated by quorum sensing networks, that is chemical communication systems that facilitate intercellular coordination of metabolic activity. Notably, the production of biosurfactants has been shown to be induced under nutrient limitation, implicating this phenotype as a dispersal mechanism (Manresa et al., 1991). Additionally, biosurfactants are well-characterized to be required for dispersal from biofilms, further solidifying their role as microbial dispersal agents (Boles et al., 2005; Davey et al., 2003). Experimental addition of surfactants to liquid solutions significantly alters bubble rupture kinetics by prolonging bubble lifespan prior to rupture, resulting in a thinner bubble dome that generates smaller film drops upon collapse. This in turn results in a more forceful rupture event that both generates smaller particles and ejects them further from the rupture point (Ke et al., 2017., Poulain and Bourouiba, 2018). Additionally, *E. coli* cells positioned throughout a bubble film have been directly observed using interferometry imaging, further demonstrating the potential importance of bubble rupture dynamics rather than fluid properties alone as a crucial regulator of microbial emissions (Poulain and Bourouiba, 2018). Notably, these surfactant-modulated bubble rupture events also generate bioaerosols from solid surfaces when air becomes entrapped between a microbially-colonized surface and an impacting droplet of rainwater. Raindrop impacts on solid surfaces have been demonstrated to be robust dispersal sources for bacterial bioaerosols (Joung et al., 2017), and biosurfactant-producing bacteria and fungi are known to colonize plant and other surfaces subjected to rainfall (Burch et al., 2014; Luft et al., 2020). Taken together, these findings suggest microbial biosurfactant secretion may enhance microbial uplift by altering bubble burst dynamics in a manner that specifically favors bioaerosol generation across diverse environmental contexts from both solid and liquid surfaces. Further exploration into the interplay between microbial metabolites and bioaerosol generation is needed, perhaps examining traits promoting biosurfactant secretion and cell entrainment in bubble films as a next step.

### Cellular buoyancy modulation positions cells at the air–water interface

Regulation of cellular buoyancy in aquatic systems may influence aerosolization potential by positioning cells at the air-water interface (Blanchard and Syzdek, 1982). Gas vesicles are protein-bound, gas-filled nanostructures that allow certain bacteria and archaea to control their vertical position within a water column by modulating cellular buoyancy, which is believed to be used for nutrient acquisition (Walsby, 1994). These structures can facilitate accumulation of cells near the air-water interface of the sea surface microlayer (Ramsay and Salmond, 2012); buoyancy control may also increase the likelihood of entrainment during processes known to drive microbial aerosolization such as bubble bursting, wave action, or sea spray formation (Blanchard and Syzdek, 1982). As a secondary ecological dispersal benefit, the influence of gas vesicles on colony buoyancy and aggregate structure may indirectly affect aerodynamic particle size and transport once cells or aggregates are aerosolized. These features may enhance both the likelihood of initial entrainment and airborne persistence during short- to mid-range transport, potentially complementing survival traits such as desiccation resistance. From an evolutionary perspective, traits like gas vesicles may therefore serve dual roles: optimizing access to light and nutrients in aquatic systems, while also indirectly promoting aerial dispersal.

### EPS production and aggregation influences uplift in marine and terrestrial systems

Cell aggregation is a fundamental microbial adaptation well known for its role in survival and metabolic efficiency (Flemming et al., 2016) yet it may also play a critical role in microbial uplift and dispersal. Aggregates of microbial cells are typically held together by an extracellular polymeric substance (EPS) matrix composed of extracellular DNA, proteins, and polysaccharides (McDougald et al., 2012). These multicellular assemblages are termed biofilms when surface-attached and aggregates when unattached, although both forms share phenotypic traits (Kragh et al., 2023). Unlike active biofilm dispersal, where EPS is degraded (Flemming and Wingender, 2010) or production is downregulated to release cells (Barraud et al., 2006; Rollet et al., 2009), the presence of EPS and aggregate formation may result in the preferential uplift of microbial cells due to favorable increases in particle size, density, and stability. This is consistent with the finding that most airborne cells are attached to particulates or other cells and rarely observed as individual cells when aloft (Ruiz-Gil et al., 2020).

The role of EPS in microbial uplift is documented in marine environments, where the sea surface microlayer (SML) is enriched in EPS components (e.g., lipids, proteins, and polysaccharides) (Liss and Duce, 1997; Cunliffe and Murrell, 2009). EPS has also been identified as a common constituent of marine aerosols (Bigg and Leck, 2008; Leck et al., 2013). Similar surface films have been observed in lakes, ponds, rivers, and estuaries (Cai, 2020), indicating that EPS-rich interfaces are found across various aquatic systems. Much of the aggregates in the SML consist of bacteria and EPS derived from phytoplankton (Passow, 2002). These aggregates are buoyant (Cai, 2020), enabling microbes to persist at the air-water interface, facilitating their uplift into the atmosphere through mechanical processes as previously mentioned. EPS may further promote atmospheric uplift and dispersal by enhancing the structural stability of the aggregates (Persat et al., 2015; Flemming et al., 2016) and water droplets upon uplift. The association between EPS and preferential aggregation has potential implications for human health as antibiotic resistance genes have been routinely observed as more abundant in aggregated and particle-attached microbial communities than free-living cells in freshwater systems (Zhu et al., 2017; Proia et al., 2018; Ash et al., 2002). Experimental evidence also supports a central role for aggregation in aerosolization efficiency. Chamber studies have found that cells suspended organic-rich spent culture medium aerosolize more effectively than those in saline solutions (Dybwad and Skogan, 2017), an effect possibly linked to enhanced aggregation in the presence of extracellular material, though the mechanism for this disparity has not been investigated.

Similar to aquatic systems, EPS-mediated uplift likely influences microbial uplift in terrestrial environments. Microbial aggregation with surface soil, dust, and other particles can comparably alter effective particle size and stability (Després et al., 2012; Smets et al., 2016; Joung et al., 2017), thereby enhancing wind-driven uplift while reducing fragmentation during mechanical disruption. Furthermore, EPS accumulation on leaf surfaces can promote the projection of cells that extend away from the leaf surface (Morris et al., 1997), thereby increasing cell exposure to mechanical removal. Smaller aggregates of microbial cells within terrestrial biofilms at air-surface interfaces may also be passively uplifted. Although the uplift is passive, microbial cells within biofilms can regulate their adhesive forces, with EPS playing a central role in mediating cell–cell adhesion. Reported adhesive forces fall within ranges that are susceptible to uplift by wind or abrasion (Morris and Monier, 2003), and this is thought to be a potential mechanism by which plant-associated bacteria are uplifted and dispersed (Fang et al., 2000; Auerbach et al., 2000). Therefore, while atmospheric uplift may not have been a primary evolutionary driver of microbial EPS production and aggregation, it has become a significant secondary benefit and may represent a more recent evolutionary pressure.

### Biofilm dispersion and possible uplift

Plants have been studied as a major source of airborne microorganisms since the 1980s (Lindemann et al., 1982). Epiphytic bacteria typically exist as biofilms or multicellular aggregates on plant surfaces (Yaron and Römling, 2014), and the majority of viable airborne bacteria are similarly found attached to particulates or other cells rather than as free-floating individual cells (Montero et al., 2016; Després et al., 2012; Smets et al., 2016). A direct transfer of leaf-surface aggregates into airborne aggregates has not been demonstrated, but uplift of epiphytic aggregates or biofilm fragments is plausible. Dispersal of bacterial biofilms is a highly regulated process marking the end of a biofilm’s life cycle, typically triggered by a decrease in intracellular cyclic di-GMP (c-di-GMP) levels, leading differentiated motile cells to release from established biofilms to colonize new surfaces (McDougald et al., 2012). Both environmental and physiological cues including changes in nutrient availability or temperature, hypoxia, or low nitric oxide levels can modulate c-di-GMP concentrations in bacterial models and have been linked directly to dispersal initiation (McDougald et al., 2012). Whether biofilm dispersal on leaves might coincide with increased aerosolization remains unknown, though such coupling could in principle benefit pathogens that rely on aerial transmission. *Pseudomonas syringae*, for example, forms biofilms and is known to disperse via aerosols during its life cycle (Morris et al., 2008), and its ice-nucleating phenotype has been suggested to be linked to enhanced aerosolization in lab studies (Pietsch et al., 2015), furthering questions around how physiological traits may influence uplift. Coupling biofilm dispersal with aerosolization could enable conditional dispersal that links physiological state to opportunities for atmospheric transport, which in turn, suggests that biofilm regulatory pathways could be indirectly shaped by selection for improved dispersal efficiency.

### Cell size may not be a primary determinant of uplift

Cell size may influence microbial aerosolization primarily by shaping entrainment constraints rather than acting as a strict selective determinant of uplift. Bioaerosol simulations and traits inferred from genomic identification have shown that smaller eukaryotic organisms (<20 μm) are more readily uplifted and may remain airborne longer (Wilkinson et al., 2011; Echenique-Subiabre et al., 2025), yet larger cells can also achieve long-range transport under favorable conditions (Galbán et al., 2021). Additionally, sea spray aerosol investigations demonstrate that sufficiently large droplets can entrain organisms spanning a wide size range from ~ 2 to 500 μm (Sinnett et al., 2025; Wiśniewska et al., 2022),further supporting that cell size alone does not determine aerosolization probability. Chamber studies have found no correlation between preferential aerosolization and cell size or shape across five species tested with two nebulization methods, instead theorizing that bacterial properties caused the enrichment in air (Perrott et al., 2017). Collectively, this evidence suggests that while cellular size influences aerodynamic behavior, the ability to associate with carrier particles or form aggregates is likely more consequential in uplift, underscoring the need for additional direct measurements of microbial structure at the particle level with techniques such as flow cytometry or fluorescent microscopy to examine correlations with organisms’ size.

### Electrical charge may influence uplift behavior

No direct evidence of cell surface charge altering aerosolization likelihood has been found, though uplift could feasibly be influenced by electrostatic repulsion or adhesion interactions with charged surfaces. Airborne microorganisms can carry non-negligible electrical charge that can vary from tens to thousands of elementary charges per cell, arising from the surface-particle collisions during mechanical dispersion and cell envelope characteristics (Mainelis et al., 2001; Simon et al., 2015). Both Gram-negative and Gram-positive bacteria carry a net negative surface charge largely generated by ionizable amino, carboxyl, and phosphate groups arising from outer membrane proteins and lipopolysaccharides (Mainelis et al., 2001; van der Wal et al., 1997). In some Gram-positive species, phosphate-rich teichoic acids on the cell surface confer particularly negative surface charges, though electrostatic properties vary widely across Gram-positive and Gram-negative taxa and environmental conditions (Jiang et al., 2004; Wilhelm et al., 2021; Cremin et al., 2020). Electrostatic forces are known to affect aerosol transport and deposition as charged bacteria show enhanced attraction to oppositely charged surfaces, so much so that this effect has been exploited for bioaerosol sampling techniques (Meschke et al., 2009; Hong et al., 2021). Simulation studies have shown that naturally occurring electric fields above thunderstorms may provide sufficient force to uplift charged microbial particles into the high atmosphere (Dehel et al., 2008). Surface charge is a key property of a microbe’s cell surface that mediates adhesion and aggregation interactions with other cells and surfaces, and is therefore a trait subject to ecological selection through its effect on surface colonization and biofilm formation (Bos et al., 1999). Any potential influence of surface charge on aerosolization may be a secondary consequence of this (or other) traits’ relevance with surface-associated lifecycles.

### Microbial airborne dispersal via animal host relationships

Animals act as active vectors of microbial uplift wherein microbial traits enabling attachment or detachment from their hosts could provide enhanced aerosolization potential distinct to the air or environment due to the specialized niche. Flies (Nayduch et al., 2023), balloon-producing spiders (Morley and Robert, 2018), and birds (Coughlan et al., 2017) among other examples can disperse attached microorganisms over distances similar to or exceeding passive atmospheric dispersal. Animal vectors provide protection to fungal propagules during dispersal while within fur, feathers, or mouths (Korkmaz et al., 2025). Rodents can facilitate active dispersal equally efficiently as passive wind dispersal (Borgmann-Winter et al., 2023). Microbes capable of surviving digestion can be released in feces or frass, after which drying and mechanical disturbance of the excrement can promote aerosolization within this secondary matrix. In marine ecosystems, exhaled respiratory plumes from breaching whales can selectively entrain microorganisms in the air which are associated primarily with the host rather than surrounding seawater (Apprill et al., 2017). These examples underscore that traits linked to attachment and/or detachment to surfaces are influential in biologically hosted or generated aerosols. Thus, animal-mediated dispersal may also act as a selective process, albeit likely a small one, favoring certain microbial attributes.

## Contexts of microbial uplift

### Sources of continuous microbial uplift to the atmosphere

**Figure 3:**
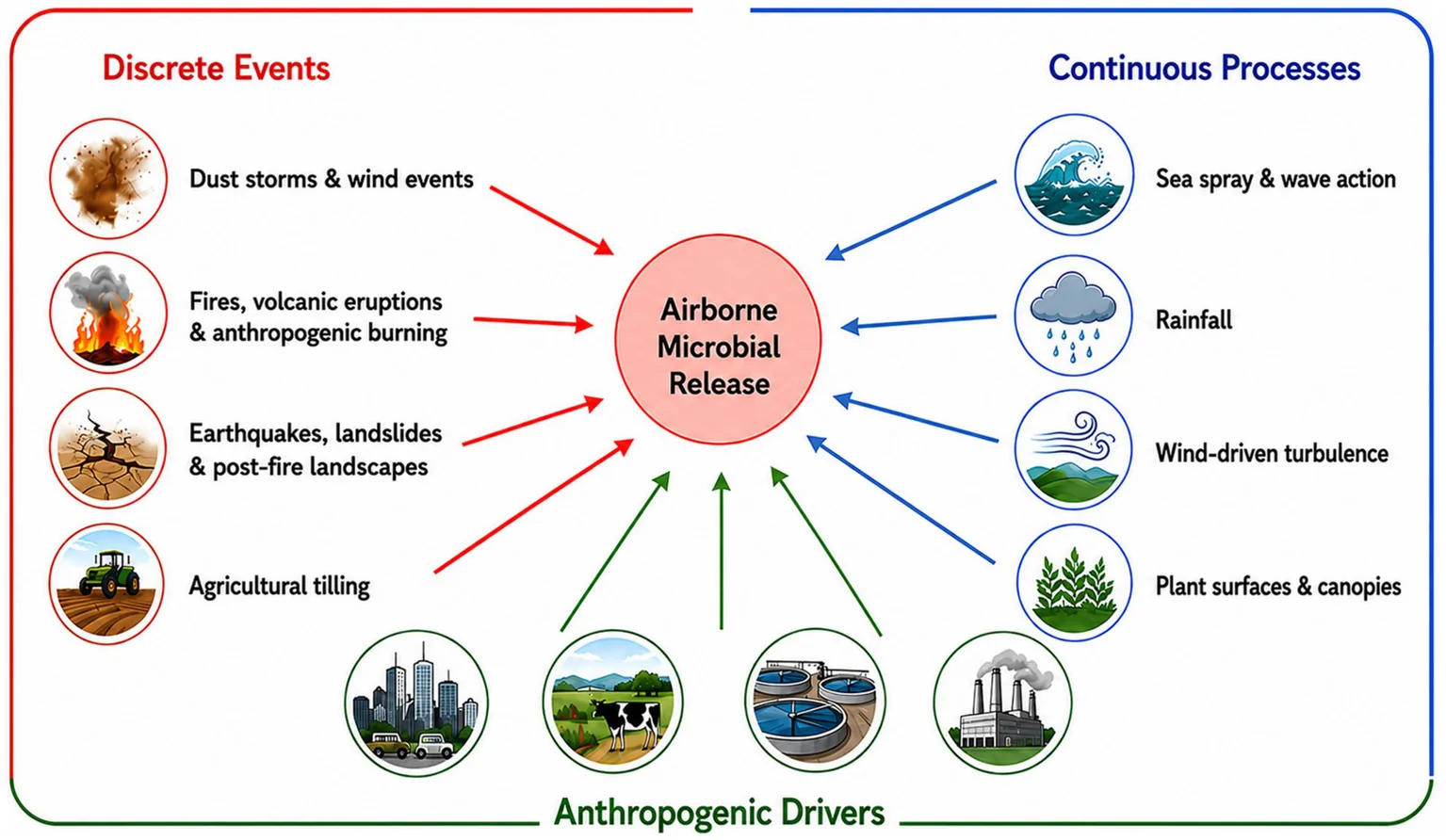
Microorganisms enter the atmosphere through interconnected discrete, continuous, and anthropogenic drivers across ecosystems wherein entrainment in the atmosphere is likely impacted by microbial traits. Continuous processes are persistent, operating at local to regional scales, discrete processes are high-energy disturbances that produce rapid pulses that may include significant numbers of microbes and long dispersal distances, and anthropogenic processes intensify and modify atmospheric emissions of microorganisms.

While large-scale discrete events such as dust storms or wildfires are recognized as significant contributors to microbial emissions, the continuous transfer of microorganisms from surfaces to the atmosphere is a critical aspect of biological dispersal and global biogeochemical cycles. Continuous physical uplift processes using biological mechanisms described previously in natural environments include wind-driven wave breaks over bodies of water generating sea spray aerosols, rainfall, and wind-driven turbulence over surfaces and plants (Figure 3). Studies examining how environmental factors such as temperature, relative humidity, rainfall, and wind impact aerosolization have focused on particular habitats like wastewater treatment plants, agricultural settings, indoor public environments, urban ecosystems, soils, etc. (Salari et al., 2025; Mengyao et al., 2025; Islam et al., 2020; Kwak et al., 2024). Due to issues with detection limits in the air, many ecosystem-based studies quantify airborne microbial concentration using culture-based approaches (Salari et al., 2025; Mengyao et al., 2025; Islam et al., 2020) which bias detection toward culturable heterotrophs, though molecular methods such as qPCR and amplicon sequencing are increasingly utilized (Fang et al., 2018; Kwak et al., 2024). A primary limitation of field measurements is the difficulty in separating uplift from post-emission survival, as sampling often occurs after atmospheric selection has already acted. Comparative spatial or temporal studies could partially address this difficulty but isolation of aerosolization processes directly is still a needed area of study.

Across studies, rainfall is observed as one of the most consistent drivers of increased aerosolization of bacteria, with particle size (Kwak et al., 2024; Ruiz-Gil et al., 2025) and soil type (Joung et al., 2017) influencing emission magnitude. Estimates of rainfall contribution to bioaerosols range from 1.6–25% of the total amount of bacteria emitted from soils (Ruiz-Gil et al., 2025). In contrast, the effects of other parameters such as temperature, humidity (RH), ionic strength, and source of bacterial load are highly context dependent, varying with organism identity, source, and particle characteristics (Chai et al., 2023). Various environmental parameters have a greater influence for different organisms and for different locations, with humidity and source bacterial load appearing to have a higher influence than other parameters in most contexts. For example, humidity can either suppress or enhance aerosolization of different taxa (Chai et al., 2023; Alsved et al., 2020).

Precipitation is a major driver of bioaerosol generation and deposition with droplet size linked to varied mechanisms of microbial dispersal. Falling raindrops commonly facilitate microbial and fungal deposition from the atmosphere, but also produce short, intense mechanical shaking or air movements that can exceed 100 m.p.h., and dislodge spores from dry vegetation (coined the tap and puff mechanism) (Hirst and Stedman, 1963). In this mechanism of microbial dispersal, efficiency depends on both the size of the spore and water droplet. Studies examining dispersal of plant pathogens *Phytophthora cactorum* and *Colltotrichum acutatum* showed that larger droplets increased the number of spores disseminated and the flight distance, likely through increased ejection velocity and kinetic energy (Madden, 1997). Raindrop impacts on leaves, stems, and soils can further mobilize microbes via lofting, though transport is typically localized and moderated by ground cover and plant canopy (Van Stan II et al., 2020; Madden, 1997). Beyond rainfall, precipitation in the form of dew can initiate spore release by passive or active methods by wetting fungal fruiting bodies. For example, Iliff et al. (2022) documented a unique mechanism whereby the wheat pathogen, *Epicoccum tritici,* is dispersed by dew droplets enabling release from the tips of wheat awn hairs to neighboring stomatal ridges by changes in pressure and surface wettability. Spores then agglomerated into dry clusters via evaporation and were passively dispersed via wind or rain splash. Similarly, moisture, like dew droplets, enables the development of spores that can then be forcibly discharged via turgor pressure or surface tension. In the Basidiomycete fungi, a fluid droplet called Buller’s drop forms over the spore surface and continues to grow until the spores are forcibly discharged from gills, spines, or tube surfaces. Fluid movement is powered by surface tension that catapult the spores (Turner and Webster, 1991).

### Microbial uplift during discrete events

Episodic physical disturbances, often in the form of extreme weather events or acute anthropogenic perturbations, produce abrupt pulses in atmospheric microbial abundance by rapidly aerosolizing organisms (Figure 3). The sudden input of energy during these events can overcome microbial adhesion forces, resulting in the uplift of particulate matter and attached microbes or microbial cells (Alsved et al., 2020). Microbial traits allowing attachment to particulates or easier detachment and/or persistence from surfaces hit by uplifting forces like wind could be evolutionarily beneficial in facilitating dispersal during such events. Dust storms are the most extensively studied discrete aerosolization events; capable of uplifting particulates and associated microorganisms to enable dispersal across oceans and at continental scales (Griffin, 2007). As microorganisms are primarily transported as aggregates with particulates rather than as individual cells, traits promoting aggregation or adherence to particles such as EPS production may disproportionately enhance an organism’s uplift during high-energy events. The ability to aggregate to particles or other traits may enhance microbial uplift in other episodic perturbations or over shorter dispersal scales.

In post-fire landscapes (Yu and Ginoux, 2022), earthquake-associated dust liberation, landslides (Schneider et al., 1997), and agricultural tilling (Schlatter et al., 2018), microorganisms attached to soil, ash, or other particulate matter are favored for uplift. Microbes are also uplifted within large quantities of smoke and ash injected into the atmosphere via thermally driven disturbances such as wildfires, anthropogenic burning, and volcanic eruptions, with viable cells and fungal spores detected from these sources (Witt et al., 2017; Moore et al., 2021; Mims and Mims, 2004; Wei et al., 2019). Although terrestrial ecosystems represent the dominant source of airborne microbes, episodic events over marine environments also contribute substantially to bioaerosols (Cao et al., 2024; Griffin et al., 2017).

Hurricanes, tsunamis, and increased wave-breaking activity dramatically increase bubble bursting at air-water interfaces (Zhou et al., 2025), increasing aerosolization of organisms enriched in the sea surface microlayer. Enrichment in this layer, and thus preferential aerosolization, is influenced by microbial traits such as cell hydrophobicity and cell buoyancy via internal gas vesicles. While hurricanes mainly aerosolize large quantities of marine microorganisms, terrestrial-, human-, and animal-associated bacteria (e.g., *Escherichia* and *Streptococcus*) can be uplifted as well when passing over populated areas (DeLeon-Rodriguez et al., 2013). Together, these processes highlight that discrete disturbance events are powerful mechanisms for mobilizing and redistributing microbes through the atmosphere, while intrinsic microbial traits enhance an organism’s ability to exploit these transient opportunities for uplift. Consequently, high-energy episodic events may function less as indiscriminate aerosolization/dispersal mechanisms and more as ecological filters by selecting for microbial phenotypes that enhance their association with particulates, sea surface enrichment, or perhaps detachment.

### Anthropogenic impacts on uplift

Anthropogenic environments can create novel and intensified drivers of microbial aerosolization and dispersion by modifying airflow, increasing particle emissions, and generating concentrated sources of microorganisms (Figure 3) while potentially making the atmosphere more habitable as a whole (Ginn et al., 2021; Archer and Pointing, 2020). In cities, urban infrastructure alters air movement and creates microenvironments that influence aerosol dispersion (Huang et al., 2021); higher population density can amplify opportunities for airborne transmission of microorganisms; and urban air pollution can influence microbial transmission dynamics (Franchitti et al., 2022). Agricultural activities, including crop harvesting and livestock production, emit large quantities of particulates and airborne microorganisms (Suski et al., 2018; Zhao et al., 2014). Similarly, wastewater treatment processes produce bioaerosols and aerosolize enteric bacteria through mechanical agitation/aeration and bubble bursts. Bacterial source load has been found to influence abundance of microorganisms in aerosols, with studies on wastewater plants and farms finding higher concentrations of bioaerosols over areas with higher microbial load (Dungan, 2010). Beyond direct emissions, anthropogenic activities that drive land use change and climate warming can indirectly enhance microbial uplift by increasing dust mobilization and deposition to oceans, stimulating marine productivity which then feeds back into increased aerosolization of marine microorganisms (Pointing and Belnap, 2014). Although anthropogenic activities have demonstrable effects on microbial uplift mechanisms and community properties (Jiang et al., 2022), these mechanisms are relatively new in evolutionary terms. Microorganisms therefore may not have had sufficient time to evolve specific adaptations to them; instead, anthropogenic forces may enhance traits typically linked to natural dispersal drivers such as wind.

## Conclusions and future research priorities

Though aerosolization of microorganisms is often thought of as a passive mechanism of dispersal, physical processes do not fully explain uplift behavior. Instead, microbial aerosolization is best understood as an interaction between biological traits and the environment in which physical forcing intersects with microbial features to determine the probability of uplift. We have highlighted traits that increase residence time at air-water interfaces and exposed surfaces, promote ejection via bubble bursting, and facilitate sloughing into the atmosphere as key mechanisms for increased aerosolization, with more traits likely to be discovered when assessed from this new lens. Evidence across laboratory, field, and sequence-based studies indicates that uplift acts as an ecological filter shaping which organisms disperse via air. Collectively, these studies support framing aerosolization as an ecologically and evolutionarily relevant filter, the understanding of which would greatly improve predictive understanding of microbial uplift and dispersal across environments.

Many microbial traits that influence aerosolization likely evolved as adaptations for other ecological functions such as aggregation or environmental persistence, but may confer secondary dispersal advantages once organisms encounter aerosol-generating conditions. Therefore, traits including cell surface hydrophobicity, EPS production, and others discussed in this review may incidentally increase the probability of atmospheric entrainment by promoting associated with interfaces or particles subjected to uplift processes. Traits relevant to aerosolization success may function as exaptations: features selected for in one ecological context that provide a consequential advantage in another context (Larson et al., 2013). Atmospheric dispersal increases access to new habitats and host colonization opportunities over varied scales so these incidental benefits may act as secondary selective pressures favoring phenotypes that enhance aerosolization probability. Such a framework positions microbial uplift not as a purely passive, stochastic process but as an ecological filter through which pre-existing microbial traits can be evolutionarily valuable in atmospheric transmission.

Mechanistic evidence for trait-modulated aerosolization remains limited in support of this conceptual framework. Chamber studies to-date suggest microbial surface properties can influence aerosolization efficiency, but results are inconsistent and based on a small number of tested strains and experimental conditions (Perrott et al., 2017). Controlled chamber experiments therefore represent a crucial next step to resolving the biological determinants of uplift. Experiments comparing isogenic mutants, phenotypic variants, or closely related organisms under standardized aerosolization conditions could isolate the effects of candidate traits and would provide valuable insight in quantifying trait-based controls on aerosolization. For example, controlled wind tunnel chambers containing mixed microbial communities inoculated on a surface could employ genomic sequencing of air and source material to identify organisms that are consistently differentially aerosolized. Sparging Liquid Aerosol Generator (SLAG) nebulizers (CH Technologies, Westwood, NJ, USA) are used to imitate bubble burst aerosolization events, which can be used to quantitatively assess the impact of traits such as hydrophobicity or biosurfactant secretion on uplift potential.

Integrating ever improving metatranscriptomics techniques into controlled aerosolization experiments offers opportunities to link environmental forcing with active microbial physiology (Kraus et al., 2025). Metatranscriptomic data from clouds have shown direct, active interaction with the environment (Amato et al., 2017), while chamber based transcriptomic studies have demonstrated that messenger RNA provides a near–real-time signal of microbial responses to aerosolization and atmospheric stressors, capturing rapid shifts in metabolism that cannot be inferred from genomic potential alone (Kraus et al., 2025). Coordinated sampling following expression changes in aerosols would allow identification of differentially expressed genes including previously uncharacterized genes and proteins that may play critical roles in aerosolization but lack current functional annotation. This is particularly valuable in aeromicrobiology, where a substantial proportion of detected sequences remain poorly described, and where trait discovery has historically been constrained by culture-based or candidate-gene approaches (Tastassa et al., 2024; Huang et al., 2024).

The role of aggregation between bioaerosols and particulates in uplift could be elucidated by microscopy experiments coupled with size-fractionated aerosol sampling for genomic identification of microorganisms associated with different particle sizes. Further coupling of trait-centric chamber studies with field source tracking and atmospheric flux measurements could allow direct testing of phenotypes favorable to promoting aerosolization across varied environments. Delineating such mechanistic links would advance our understanding of microbial uplift as an ecological process and filter, thus improving our ability to predict microbial dispersal, disease exposure pathways, and ecosystem connectivity through the atmosphere.

## References

[ref1] Aller J. Y. Kuznetsova M. R. Jahns C. J. Kemp P. F. (2005). The sea surface microlayer as a source of viral and bacterial enrichment in marine aerosols. J. Aerosol Sci. 36, 801–812. doi: 10.1016/j.jaerosci.2004.10.012, 38826717

[ref2] Alsved M. Bourouiba L. Duchaine C. Löndahl J. Marr L. C. Parker S. T. . (2020). Natural sources and experimental generation of bioaerosols: challenges and perspectives. Aerosol Sci. Technol. 54, 547–571. doi: 10.1080/02786826.2019.1682509

[ref3] Amato P. Joly M. Besaury L. Oudart A. Taib N. Moné A. I. . (2017). Active microorganisms thrive among extremely diverse communities in cloud water. PLoS One 12:e0182869. doi: 10.1371/journal.pone.0182869, 28792539 PMC5549752

[ref4] Angenent L. T. Kelley S. T. St Amand A. Pace N. R. Hernandez M. T. (2005). Molecular identification of potential pathogens in water and air of a hospital therapy pool. Proc. Natl. Acad. Sci. USA 102, 4860–4865. doi: 10.1073/pnas.0501235102, 15769858 PMC555732

[ref5] Apprill A. Miller C. A. Moore M. J. Durban J. W. Fearnbach H. Barrett-Lennard L. G. (2017). Extensive core microbiome in drone-captured whale blow supports a framework for health monitoring. mSystems 2, e00119–17. doi: 10.1128/mSystems.00119-17, 29034331 PMC5634792

[ref6] Archer S. D. J. Lee K. C. Caruso T. Alcami A. Araya J. G. Cary S. C. . (2023). Contribution of soil Bacteria to the atmosphere across biomes. Sci. Total Environ. 871:162137. doi: 10.1016/j.scitotenv.2023.162137, 36775167

[ref7] Archer S. D. J. Pointing S. B. (2020). Anthropogenic impact on the atmospheric microbiome. Nat. Microbiol. 5, 229–231. doi: 10.1038/s41564-019-0650-z, 31992892

[ref8] Ash R. J. Mauck B. Morgan M. (2002). Antibiotic resistance of gram-negative Bacteria in Rivers, United States. Emerg. Infect. Dis. 8, 713–715. doi: 10.3201/eid0807.010264, 12095440 PMC2730334

[ref9] Auerbach I. D. Sorensen C. Hansma H. G. Holden P. A. (2000). Physical morphology and surface properties of unsaturated *Pseudomonas Putida* biofilms. J. Bacteriol. 182, 3809–3815. doi: 10.1128/jb.182.13.3809-3815.2000, 10850998 PMC94554

[ref10] Baas Becking L. G. M. (1934). Geobiologie of Inleiding Tot de Milieukunde. The Hague (Den Haag), Netherlands: WP Van Stockum & Son.

[ref11] Barraud N. Hassett D. J. Hwang S.-H. Rice S. A. Kjelleberg S. Webb J. S. (2006). Involvement of nitric oxide in biofilm dispersal of *Pseudomonas Aeruginosa*. J. Bacteriol. 188, 7344–7353. doi: 10.1128/JB.00779-06, 17050922 PMC1636254

[ref12] Bigg E. K. Leck C. (2008). The composition of fragments of bubbles bursting at the ocean surface. J. Geophys. Res. Atmos. 113:D11209. doi: 10.1029/2007JD009078

[ref13] Blanchard D. C. Syzdek L. D. (1982). Water-to-air transfer and enrichment of Bacteria in drops from bursting bubbles. Appl. Environ. Microbiol. 43, 1001–1005. doi: 10.1128/aem.43.5.1001-1005.1982, 16346001 PMC244176

[ref14] Boles B. R. Thoendel M. Singh P. K. (2005). Rhamnolipids mediate detachment of *Pseudomonas Aeruginosa* from biofilms. Mol. Microbiol. 57, 1210–1223. doi: 10.1111/j.1365-2958.2005.04743.x, 16101996

[ref15] Borgmann-Winter B. W. Stephens R. B. Anthony M. A. Frey S. D. D’ Amato A. W. Rowe R. J. (2023). Wind and small mammals are complementary fungal dispersers. 104:e4039. doi: 10.1002/ecy.4039 36960918

[ref16] Bos R. van der Mei H. C. Busscher H. J. (1999). Physico-chemistry of initial microbial adhesive interactions – its mechanisms and methods for study. FEMS Microbiol. Rev. 23, 179–230. doi: 10.1111/j.1574-6976.1999.tb00396.x 10234844

[ref17] Burch A. Y. Zeisler V. Yokota K. Schreiber L. Lindow S. E. (2014). The hygroscopic biosurfactant syringafactin produced by *Pseudomonas syringae* enhances fitness on leaf surfaces during fluctuating humidity. Environ. Microbiol. 16, 2086–2098. doi: 10.1111/1462-2920.12437, 24571678

[ref18] Burrows S. M. Butler T. Jöckel P. Tost H. Kerkweg A. Pöschl U. . (2009). Bacteria in the global atmosphere – part 2: modeling of emissions and transport between different ecosystems. Atmos. Chem. Phys. 9, 9281–9297. doi: 10.5194/acp-9-9281-2009

[ref19] Cai Y.-M. (2020). Non-surface attached bacterial aggregates: a ubiquitous third lifestyle. Front. Microbiol. 11:557035. doi: 10.3389/fmicb.2020.557035, 33343514 PMC7746683

[ref20] Cao Y. Xu T. Yu X. Hua Z. Liu H. Gu W. . (2024). Fungal aerosols over the Northwest Pacific to Arctic Ocean: the influence of air mass and environmental factors. Atmos. Environ. 337:120776. doi: 10.1016/j.atmosenv.2024.120776

[ref21] Chai A. Yuan L. Li X. Li L. Shi Y. Xie X. . (2023). Effect of temperature and humidity on dynamics and transmission of *Pseudomonas amygdali* pv. *Lachrymans* aerosols. Front. Plant Sci. 14:1087496. doi: 10.3389/fpls.2023.1087496, 36818834 PMC9936812

[ref22] Coughlan N. Kelly T. Davenport J. Jansen M. (2017). Up, up and away: bird-mediated ectozoochorous dispersal between aquatic environments. Freshw. Biol. 62, 631–648. doi: 10.1111/fwb.12894

[ref23] Cremin K. Jones B. A. Teahan J. Meloni G. N. Perry D. Zerfass C. . (2020). Scanning ion conductance microscopy reveals differences in the ionic environments of gram-positive and negative Bacteria. Anal. Chem. 92, 16024–16032. doi: 10.1021/acs.analchem.0c03653, 33241929

[ref24] Cunliffe M. Engel A. Frka S. Gašparović B. Guitart C. Murrell J. C. . (2013). Sea surface microlayers: a unified physicochemical and biological perspective of the air–ocean interface. Prog. Oceanogr. 109, 104–116. doi: 10.1016/j.pocean.2012.08.004

[ref25] Cunliffe M. Murrell J. C. (2009). The sea-surface microlayer is a gelatinous biofilm. ISME J. 3, 1001–1003. doi: 10.1038/ismej.2009.69, 19554040

[ref26] Davey M. E. Caiazza N. C. O’Toole G. A. (2003). Rhamnolipid surfactant production affects biofilm architecture in *Pseudomonas Aeruginosa* PAO1. J. Bacteriol. 185, 1027–1036. doi: 10.1128/jb.185.3.1027-1036.2003, 12533479 PMC142794

[ref27] Dehel T. Lorge F. Dickinson M. (2008). Uplift of microorganisms by electric fields above thunderstorms. J. Electrost. 66, 463–466. doi: 10.1016/j.elstat.2008.04.014

[ref28] DeLeon-Rodriguez N. Lathem T. L. Rodriguez-R L. M. Barazesh J. M. Anderson B. E. Beyersdorf A. J. . (2013). Microbiome of the Upper troposphere: species composition and prevalence, effects of tropical storms, and atmospheric implications. Proc. Natl. Acad. Sci. 110, 2575–2580. doi: 10.1073/pnas.1212089110, 23359712 PMC3574924

[ref29] Dervaux J. Mejean A. Brunet P. (2015). Irreversible collective migration of Cyanobacteria in eutrophic conditions. PLoS One 10:e0120906. doi: 10.1371/journal.pone.0120906, 25799424 PMC4370732

[ref30] Després V. R. Huffman J. A. Burrows S. M. Hoose C. Safatov A. S. Buryak G. . (2012). Primary biological aerosol particles in the atmosphere: a review. Tellus Ser. B Chem. Phys. Meteorol. 64:15598. doi: 10.3402/tellusb.v64i0.15598

[ref31] Dressaire E. Yamada L. Song B. Roper M. (2016). Mushrooms use convectively created airflows to disperse their spores. Proc. Natl. Acad. Sci. 113, 2833–2838. doi: 10.1073/pnas.1509612113, 26929324 PMC4801285

[ref32] Dungan R. S. (2010). Board-invited review: fate and transport of bioaerosols associated with livestock operations and manures. J. Anim. Sci. 88, 3693–3706. doi: 10.2527/jas.2010-3094, 20622180 PMC7109640

[ref33] Dybwad M. Skogan G. (2017). Aerobiological stabilities of different species of gram-negative Bacteria, including well-known biothreat simulants, in single-cell particles and cell clusters of different compositions. Appl. Environ. Microbiol. 83, e00823–e00817. doi: 10.1128/AEM.00823-17, 28687646 PMC5583493

[ref34] Echenique-Subiabre I. Jackrel S. L. McCarren J. James C. C. Perez-Coronel E. Tran C. . (2025). Traits determine dispersal and colonization abilities of microbes. Appl. Environ. Microbiol. 91:e0205524. doi: 10.1128/aem.02055-24, 39976438 PMC11921345

[ref35] Fahlgren C. Gómez-Consarnau L. Zábori J. Lindh M. V. Krejci R. Mårtensson E. M. . (2015). Seawater Mesocosm experiments in the Arctic uncover differential transfer of marine Bacteria to aerosols. Environ. Microbiol. Rep. 7, 460–470. doi: 10.1111/1758-2229.12273, 25682947

[ref36] Fang H. H. P. Chan K.-Y. Xu L.-C. (2000). Quantification of bacterial adhesion forces using atomic force microscopy (AFM). J. Microbiol. Methods 40, 89–97. doi: 10.1016/S0167-7012(99)00137-2, 10739347

[ref37] Fang Z. Guo W. Zhang J. Lou X. (2018). Influence of heat events on the composition of airborne bacterial communities in urban ecosystems. Int. J. Environ. Res. Public Health 15:2295. doi: 10.3390/ijerph15102295, 30347662 PMC6210276

[ref38] Flemming H.-C. Wingender J. (2010). The biofilm matrix. Nat. Rev. Microbiol. 8, 623–633. doi: 10.1038/nrmicro2415, 20676145

[ref39] Flemming H.-C. Wingender J. Szewzyk U. Steinberg P. Rice S. A. Kjelleberg S. (2016). Biofilms: an emergent form of bacterial life. Nat. Rev. Microbiol. 14, 563–575. doi: 10.1038/nrmicro.2016.94, 27510863

[ref40] Franchitti E. Caredda C. Anedda E. Traversi D. (2022). Urban aerobiome and effects on human health: a systematic review and missing evidence. Atmosphere 13:1148. doi: 10.3390/atmos13071148

[ref41] Galbán S. Justel A. González S. Quesada A. (2021). Local meteorological conditions, shape and desiccation influence dispersal capabilities for airborne microorganisms. Sci. Total Environ. 780:146653. doi: 10.1016/j.scitotenv.2021.146653, 34030336

[ref42] Ginn O. Rocha-Melogno L. Bivins A. Lowry S. Cardelino M. Nichols D. . (2021). Detection and quantification of enteric pathogens in aerosols near open wastewater canals in cities with poor sanitation. Environ. Sci. Technol. 55, 14758–14771. doi: 10.1021/acs.est.1c05060, 34669386

[ref43] Griffin D. W. (2007). Atmospheric movement of microorganisms in clouds of desert dust and implications for human health. Clin. Microbiol. Rev. 20, 459–477. doi: 10.1128/cmr.00039-06, 17630335 PMC1932751

[ref44] Griffin D. W. Gonzalez-Martin C. Hoose C. Smith D. J. (2017). “Global-scale atmospheric dispersion of microorganisms,” in Microbiology of Aerosols, eds. A.-M. Delort and P. Amato (Hoboken, NJ: John Wiley & Sons, Inc).

[ref45] Hirst J. M. Stedman O. J. (1963). Dry liberation of fungus spores by raindrops. Microbiology 33, 335–344. doi: 10.1099/00221287-33-2-335 14121209

[ref46] Hong S. Kim M.-W. Jang J. (2021). Physical collection and viability of airborne Bacteria collected under electrostatic field with different sampling media and protocols towards rapid detection. Sci. Rep. 11:14598. doi: 10.1038/s41598-021-94033-7, 34272448 PMC8285527

[ref47] Huang J. Jones P. Zhang A. Hou S. S. Hang J. Spengler J. D. (2021). Outdoor airborne transmission of coronavirus among apartments in high-density cities. Front. Built Environ. 7. doi: 10.3389/fbuil.2021.666923

[ref9001] Huang Z. Yu X. Liu Q. Maki T. Alam K. Wang Y. . (2024). Bioaerosols in the atmosphere: a comprehensive review on detection methods, concentration and influencing factors. Sci. Total Environ. 912:168818. doi: 10.1016/j.scitotenv.2023.168818, 38036132

[ref48] Iliff G. J. Mukherjee R. Gruszewski H. A. Schmale III D. G. Jung S. Boreyko J. B. (2022). Phase-change-mediated transport and agglomeration of fungal spores on wheat awns. J. R. Soc. Interface 19:20210872. doi: 10.1098/rsif.2021.0872, 35582813 PMC9114972

[ref49] Islam M. A. Ikeguchi A. Naide T. (2020). Influence of temperature and humidity on the dynamics of aerosol numbers and airborne bacteria in a dairy calf house. Biosyst. Eng. 194, 213–226. doi: 10.1016/j.biosystemseng.2020.04.003

[ref50] Jiang W. Saxena A. Song B. Ward B. B. Beveridge T. J. Myneni S. C. B. (2004). Elucidation of functional groups on gram-positive and gram-negative bacterial surfaces using infrared spectroscopy. Langmuir 20, 11433–11442. doi: 10.1021/la049043+, 15595767

[ref51] Jiang X. Wang C. Guo J. Hou J. Guo X. Zhang H. . (2022). Global Meta-analysis of airborne bacterial communities and associations with anthropogenic activities. Environ. Sci. Technol. 56, 9891–9902. doi: 10.1021/acs.est.1c07923, 35785964 PMC9301914

[ref52] Joung Y. S. Ge Z. Buie C. R. (2017). Bioaerosol generation by raindrops on soil. Nat. Commun. 8:14668. doi: 10.1038/ncomms14668, 28267145 PMC5344306

[ref53] Ke W.-R. Kuo Y.-M. Lin C.-W. Huang S.-H. Chen C.-C. (2017). Characterization of aerosol emissions from single bubble bursting. J. Aerosol Sci. 109, 1–12. doi: 10.1016/j.jaerosci.2017.03.006

[ref54] Korkmaz Y. Bełka M. Blumenstein K. (2025). How cryptic animal vectors of Fungi can influence Forest health in a changing climate and how to anticipate them. Appl. Microbiol. Biotechnol. 109:65. doi: 10.1007/s00253-025-13450-0, 40088282 PMC11910412

[ref55] Kragh K. N. Tolker-Nielsen T. Lichtenberg M. (2023). The non-attached biofilm aggregate. Commun. Biol. 6:898. doi: 10.1038/s42003-023-05281-4, 37658117 PMC10474055

[ref9002] Kraus E. A. Prithiviraj B. Hernandez M. (2025). Advancing transcriptomic profiling of airborne bacteria. Appl. Environ. Microbiol. 91, e00148–25. doi: 10.1128/aem.00148-25, 40293243 PMC12093975

[ref56] Kwak N. Tsameret S. Gaire T. N. Mendoza K. M. Cortus E. L. Cardona C. . (2024). Influence of rainfall on size-resolved bioaerosols around a livestock farm. Sci. Total Environ. 954:176184. doi: 10.1016/j.scitotenv.2024.176184, 39276997

[ref57] Larson G. Stephens P. A. Tehrani J. J. Layton R. H. (2013). Exapting exaptation. Trends Ecol. Evol. 28, 497–498. doi: 10.1016/j.tree.2013.05.018, 23764257

[ref58] Leck C. Gao Q. Mashayekhy Rad F. Nilsson U. (2013). Size-resolved atmospheric particulate polysaccharides in the high summer Arctic. Atmos. Chem. Phys. 13, 12573–12588. doi: 10.5194/acp-13-12573-2013

[ref59] Lindemann J. Constantinidou H. A. Barchet W. R. Upper C. D. (1982). Plants as sources of airborne Bacteria, including ice nucleation-active Bacteria. Appl. Environ. Microbiol. 44, 1059–1063. doi: 10.1128/aem.44.5.1059-1063.1982, 16346129 PMC242148

[ref60] Liss P. S. Duce R. A. (1997). The Sea Surface and Global Change. Cambridge: Cambridge University Press.

[ref61] Luft L. Confortin T. C. Todero I. Zabot G. L. Mazutti M. A. (2020). An overview of fungal biopolymers: bioemulsifiers and biosurfactants compounds production. Crit. Rev. Biotechnol. 40, 1059–1080. doi: 10.1080/07388551.2020.1805405, 32787550

[ref62] Lymperopoulou D. S. Adams R. I. Lindow S. E. (2016). Contribution of vegetation to the microbial composition of nearby outdoor air. Appl. Environ. Microbiol. 82, 3822–3833. doi: 10.1128/AEM.00610-16, 27107117 PMC4907200

[ref63] Madden L. V. (1997). Effects of rain on splash dispersal of fungal pathogens. Can. J. Plant Pathol. 19, 225–230. doi: 10.1080/07060669709500557

[ref64] Mainelis G. Willeke K. Baron P. Reponen T. Grinshpun S. A. Górny R. L. . (2001). Electrical charges on airborne microorganisms. J. Aerosol Sci. 32, 1087–1110. doi: 10.1016/S0021-8502(01)00039-8

[ref65] Manresa M. A. Bastida J. Mercadé M. E. Robert M. Andrés C. Espuny M. J. . (1991). Kinetic studies on surfactant production by *Pseudomonas aeruginosa* 44T1. J. Ind. Microbiol. 8, 133–136. doi: 10.1007/BF01578765

[ref66] McDougald D. Rice S. A. Barraud N. Steinberg P. D. Kjelleberg S. (2012). Should we stay or should we go: mechanisms and ecological consequences for biofilm dispersal. Nat. Rev. Microbiol. 10, 39–50. doi: 10.1038/nrmicro2695 22120588

[ref67] Mengyao C. Yang L. Guohua Q. Xiaojun L. (2025). Seasonal variation and influencing factors of airborne bacterial and fungal concentrations in different indoor public environments. Sci. Rep. 15:35934. doi: 10.1038/s41598-025-21883-w, 41087637 PMC12521343

[ref68] Meschke S. Smith B. D. Yost M. Miksch R. R. Gefter P. Gehlke S. . (2009). The effect of surface charge, negative and bipolar ionization on the deposition of airborne Bacteria. J. Appl. Microbiol. 106, 1133–1139. doi: 10.1111/j.1365-2672.2008.04078.x, 19191951

[ref69] Michaud J. M. Thompson L. R. Kaul D. Espinoza J. L. Richter R. A. Xu Z. Z. . (2018). Taxon-specific Aerosolization of Bacteria and viruses in an Experimental Ocean-atmosphere Mesocosm. Nat. Commun. 9:2017. doi: 10.1038/s41467-018-04409-z, 29789621 PMC5964107

[ref70] Mims S. A. Mims F. M. (2004). Fungal spores are transported long distances in smoke from biomass fires. Atmos. Environ. 38, 651–655. doi: 10.1016/j.atmosenv.2003.10.043

[ref71] Moletta-Denat M. Bru-Adan V. Delgenes J.-P. Hamelin J. Wéry N. Godon J.-J. (2010). Selective microbial Aerosolization in biogas demonstrated by quantitative PCR. Bioresour. Technol. 101, 7252–7257. doi: 10.1016/j.biortech.2010.04.035, 20494574

[ref72] Montero A. Dueker M. E. O’Mullan G. D. (2016). Culturable bioaerosols along an urban waterfront are primarily associated with coarse particles. PeerJ 4:e2827. doi: 10.7717/peerj.2827 28028485 PMC5182991

[ref73] Moore R. A. Bomar C. Kobziar L. N. Christner B. C. (2021). Wildland fire as an atmospheric source of viable microbial aerosols and biological ice nucleating particles. ISME J. 15, 461–472. doi: 10.1038/s41396-020-00788-8, 33009511 PMC8027831

[ref74] Morley E. L. Robert D. (2018). Electric fields elicit ballooning in spiders. Curr. Biol. 28, 2324–2330.e2. doi: 10.1016/j.cub.2018.05.057, 29983315 PMC6065530

[ref75] Morris C. E. Kobziar L. N. Christner B. C. Garros C. De Vleeschouwer F. (2025). “Biological highways in the sky,” in The Dispersal of Microorganisms, Insects and Other Small Life Forms via the Atmosphere. (Versailles: Éditions Quæ).

[ref76] Morris C. E. Monier J.-M. (2003). The ecological significance of biofilm formation by plant-associated Bacteria. Annu. Rev. Phytopathol. 41, 429–453. doi: 10.1146/annurev.phyto.41.022103.134521, 12730399

[ref77] Morris C. E. Monier J.-M. Jacques M.-A. (1997). Methods for observing microbial biofilms directly on leaf surfaces and recovering them for isolation of Culturable microorganisms. Appl. Environ. Microbiol. 63, 1570–1576. doi: 10.1128/aem.63.4.1570-1576.1997, 16535579 PMC1389557

[ref78] Morris C. E. Sands D. C. Vinatzer B. A. Glaux C. Guilbaud C. Buffière A. . (2008). The life history of the plant pathogen *Pseudomonas syringae* is linked to the water cycle. ISME J. 2, 321–334. doi: 10.1038/ismej.2007.113, 18185595

[ref79] Nayduch D. Neupane S. Pickens V. Purvis T. Olds C. (2023). House flies are underappreciated yet important reservoirs and vectors of microbial threats to animal and human health. Microorganisms 11:583. doi: 10.3390/microorganisms11030583, 36985156 PMC10054770

[ref80] Passow U. (2002). Transparent exopolymer particles (TEP) in aquatic environments. Prog. Oceanogr. 55, 287–333. doi: 10.1016/S0079-6611(02)00138-6

[ref81] Perrott P. Turgeon N. Gauthier-Levesque L. Duchaine C. (2017). Preferential Aerosolization of Bacteria in bioaerosols generated in vitro. J. Appl. Microbiol. 123, 688–697. doi: 10.1111/jam.13514, 28632907

[ref82] Persat A. Nadell C. D. Kim M. K. Ingremeau F. Siryaporn A. Drescher K. . (2015). The mechanical world of Bacteria. Cell 161, 988–997. doi: 10.1016/j.cell.2015.05.005, 26000479 PMC4451180

[ref83] Pietsch R. B. David R. F. Marr L. C. Vinatzer B. Schmale D. G. III (2015). Aerosolization of two strains (ice+ and ice–) of *Pseudomonas syringae* in a Collison nebulizer at different temperatures. Aerosol Sci. Technol. 49, 159–166. doi: 10.1080/02786826.2015.1010636

[ref84] Pointing S. B. Belnap J. (2014). Disturbance to desert soil ecosystems contributes to dust-mediated impacts at regional scales. Biodivers. Conserv. 23, 1659–1667. doi: 10.1007/s10531-014-0690-x

[ref85] Poulain S. Bourouiba L. (2018). Biosurfactants change the thinning of contaminated bubbles at Bacteria-laden water interfaces. Phys. Rev. Lett. 121:204502. doi: 10.1103/PhysRevLett.121.204502, 30500232

[ref86] Proia L. Anzil A. Subirats J. Borrego C. Farrè M. Llorca M. . (2018). Antibiotic resistance along an urban river impacted by treated wastewaters. Sci. Total Environ. 628–629, 453–466. doi: 10.1016/j.scitotenv.2018.02.083 29453174

[ref87] Ramsay J. P. Salmond G. P. C. (2012). Quorum sensing-controlled buoyancy through gas vesicles: intracellular bacterial microcompartments for environmental adaptation. Commun. Integr. Biol. 5, 96–98. doi: 10.4161/cib.18532, 22482022 PMC3291326

[ref88] Rastelli E. Corinaldesi C. Dell’Anno A. Lo Martire M. Greco S. Cristina Facchini M. . (2017). Transfer of labile organic matter and microbes from the ocean surface to the marine aerosol: an experimental approach. Sci. Rep. 7:11475. doi: 10.1038/s41598-017-10563-z, 28904380 PMC5597575

[ref89] Rollet C. Gal L. Guzzo J. (2009). Biofilm-detached cells, a transition from a sessile to a planktonic phenotype: a comparative study of adhesion and physiological characteristics in *Pseudomonas Aeruginosa*. FEMS Microbiol. Lett. 290, 135–142. doi: 10.1111/j.1574-6968.2008.01415.x, 19054076

[ref90] Ruiz-Gil T. Acuña J. J. Fujiyoshi S. Tanaka D. Noda J. Maruyama F. . (2020). Airborne bacterial communities of outdoor environments and their associated influencing factors. Environ. Int. 145:106156. doi: 10.1016/j.envint.2020.106156, 33039877

[ref91] Ruiz-Gil T. Rilling J. I. Campos M. Fujiyoshi S. Maruyama F. Acuña J. J. . (2025). Airborne bacterial communities present in particulate matter (PM2.5 and PM10) samples collected during different seasons in Temuco City in Chile. Aerobiologia 41, 759–776. doi: 10.1007/s10453-025-09877-7

[ref92] Salari M. Ardakani M. A. Nasab H. Madadizadeh F. Teimouri F. Ehrampoush M. H. . (2025). Evaluation of bacterial bioaerosols and their relationship with environmental factors in the air of the wastewater treatment Plant of Industrial. Sci. Rep. 15:44827. doi: 10.1038/s41598-025-28692-1, 41462540 PMC12749427

[ref93] Schlatter D. C. Schillinger W. F. Bary A. I. Sharratt B. Paulitz T. C. (2018). Dust-associated microbiomes from dryland wheat fields differ with tillage practice and biosolids application. Atmos. Environ. 185, 29–40. doi: 10.1016/j.atmosenv.2018.04.030, 38826717

[ref94] Schneider E. Hajjeh R. A. Spiegel R. A. Jibson R. W. Harp E. L. Marshall G. A. . (1997). A Coccidioidomycosis outbreak following the Northridge, Calif, earthquake. JAMA 277, 904–908. doi: 10.1001/jama.1997.03540350054033, 9062329

[ref95] Simon X. Bau S. Bémer D. Duquenne P. (2015). Measurement of electrical charges carried by airborne Bacteria laboratory-generated using a single-pass bubbling Aerosolizer. Particuology 18, 179–185. doi: 10.1016/j.partic.2014.05.009, 38826717

[ref96] Sinnett G. Lenain L. Braham E. Chaudhry N. A. Dinasquet J. (2025). Contribution of large marine aerosols in phytoplankton dispersal. Environ. Sci. Technol. 59, 7348–7356. doi: 10.1021/acs.est.4c14473, 40179244 PMC12004932

[ref97] Smets W. Moretti S. Denys S. Lebeer S. (2016). Airborne Bacteria in the atmosphere: presence, purpose, and potential. Atmos. Environ. 139, 214–221. doi: 10.1016/j.atmosenv.2016.05.038, 38826717

[ref98] Suski K. J. Hill T. C. J. Levin E. J. T. Miller A. DeMott P. J. Kreidenweis S. M. (2018). Agricultural harvesting emissions of ice-nucleating particles. Atmos. Chem. Phys. 18, 13755–13771. doi: 10.5194/acp-18-13755-2018

[ref9003] Tastassa A. C. Sharaby Y. Lang-Yona N. (2024). Aeromicrobiology: a global review of the cycling and relationships of bioaerosols with the atmosphere. Sci. Total Environ. 912:168478–25. doi: 10.1016/j.scitotenv.2023.168478, 37967625

[ref99] Tignat-Perrier R. Dommergue A. Thollot A. Keuschnig C. Magand O. Vogel T. M. . (2019). Global airborne microbial communities controlled by surrounding landscapes and wind conditions. Sci. Rep. 9:14441. doi: 10.1038/s41598-019-51073-4, 31595018 PMC6783533

[ref100] Turner J. C. R. Webster J. (1991). Mass and momentum transfer on the small scale: how do mushrooms shed their spores? Chem. Eng. Sci. 46, 1145–1149. doi: 10.1016/0009-2509(91)85107-9

[ref101] van der Wal A. Norde W. Zehnder A. J. B. Lyklema J. (1997). Determination of the total charge in the cell walls of gram-positive bacteria. Colloids Surf. B Biointerfaces 9, 81–100. doi: 10.1016/S0927-7765(96)01340-9

[ref102] Van Stan J. T. II Morris C. E. Aung K. Kuzyakov Y. Magyar D. Rebollar E. . (2020). “Precipitation partitioning—hydrologic highways between microbial communities of the plant microbiome?” in Precipitation Partitioning by Vegetation, eds. J. T. Van Stan II, E. Gutmann, and J. Friesen (Cham: Springer International Publishing).

[ref103] Walsby A. E. (1994). Gas Vesicles. Microbiol. Rev. 58, 94–144. doi: 10.1128/mr.58.1.94-144.1994, 8177173 PMC372955

[ref104] Wei M. Xu C. Xu X. Zhu C. Li J. Lv G. (2019). Characteristics of atmospheric bacterial and fungal communities in PM2.5 following biomass burning disturbance in a rural area of North China plain. Sci. Total Environ. 651, 2727–2739. doi: 10.1016/j.scitotenv.2018.09.399 30463127

[ref105] Wilhelm M. J. Sharifian Gh M. Wu T. Li Y. Chang C.-M. Ma J. . (2021). Determination of bacterial surface charge density via saturation of adsorbed ions. Biophys. J. 120, 2461–2470. doi: 10.1016/j.bpj.2021.04.018, 33932437 PMC8390864

[ref106] Wilkinson D. M. Koumoutsaris S. Mitchell E. A. Bey I. (2011). Modelling the effect of size on the aerial dispersal of microorganisms. J. Biogeogr. 39, 89–97. doi: 10.1111/j.1365-2699.2011.02569.x, 40046247

[ref107] Wiśniewska K. A. Śliwińska-Wilczewska S. Lewandowska A. U. (2022). Airborne microalgal and cyanobacterial diversity and composition during rain events in the southern Baltic Sea region. Sci. Rep. 12. doi: 10.1038/s41598-022-06107-9, 35132131 PMC8821709

[ref108] Witt V. Ayris P. M. Damby D. E. Cimarelli C. Kueppers U. Dingwell D. B. . (2017). Volcanic Ash supports a diverse bacterial Community in a Marine Mesocosm. Geobiology 15, 453–463. doi: 10.1111/gbi.12231, 28256065 PMC5413822

[ref109] Wood S. A. Rueckert A. Cowan D. A. Cary S. C. (2008). Sources of edaphic cyanobacterial diversity in the dry valleys of eastern Antarctica. ISME J. 2, 308–320. doi: 10.1038/ismej.2007.104 18239611

[ref110] Yaron S. Römling U. (2014). Biofilm formation by enteric pathogens and its role in plant colonization and persistence. Microb. Biotechnol. 7, 496–516. doi: 10.1111/1751-7915.12186, 25351039 PMC4265070

[ref111] Yu Y. Ginoux P. (2022). Enhanced dust emission following large wildfires due to vegetation disturbance. Nat. Geosci. 15, 878–884. doi: 10.1038/s41561-022-01046-6

[ref112] Zhao Y. Aarnink A. J. A. De Jong M. C. M. Groot Koerkamp P. W. G. (2014). Airborne microorganisms from livestock production systems and their relation to dust. Crit. Rev. Environ. Sci. Technol. 44, 1071–1128. doi: 10.1080/10643389.2012.746064, 32288664 PMC7113898

[ref113] Zhou S. Salter M. Bertram T. Brito Azevedo E. Reis F. Wang J. (2025). Shoreline wave breaking strongly enhances the coastal sea spray aerosol population: climate and air quality implications. Sci. Adv. 11:eadw0343. doi: 10.1126/sciadv.adw0343, 40864708 PMC12383254

[ref114] Zhu Y.-G. Zhao Y. Li B. Huang C.-L. Zhang S.-Y. Yu S. . (2017). Continental-scale pollution of estuaries with antibiotic resistance genes. Nat. Microbiol. 2:16270. doi: 10.1038/nmicrobiol.2016.270, 28134918

